# The Pivotal Role of Chemical Modifications in mRNA Therapeutics

**DOI:** 10.3389/fcell.2022.901510

**Published:** 2022-07-13

**Authors:** Albert Liu, Xiao Wang

**Affiliations:** ^1^ Department of Chemistry, Massachusetts Institute of Technology, Cambridge, MA, United States; ^2^ Broad Institute of MIT and Harvard, Cambridge, MA, United States

**Keywords:** mRNA, chemical modifications, RNA modifications, mRNA therapeutics, mRNA vaccine, mocRNA, chimeric mRNA

## Abstract

After over a decade of development, mRNA has recently matured into a potent modality for therapeutics. The advantages of mRNA therapeutics, including their rapid development and scalability, have been highlighted due to the SARS-CoV-2 pandemic, in which the first two clinically approved mRNA vaccines have been spotlighted. These vaccines, as well as multiple other mRNA therapeutic candidates, are modified to modulate their immunogenicity, stability, and translational efficiency. Despite the importance of mRNA modifications for harnessing the full efficacy of mRNA drugs, the full breadth of potential modifications has yet to be explored clinically. In this review, we survey the field of mRNA modifications, highlighting their ability to tune the properties of mRNAs. These include cap and tail modifications, nucleoside substitutions, and chimeric mRNAs, each of which represents a component of mRNA that can be exploited for modification. Additionally, we cover clinical and preclinical trials of the modified mRNA platform not only to illustrate the promise of modified mRNAs but also to call attention to the room for diversifying future therapeutics.

## Introduction

mRNA has emerged as an important platform for gene therapies and vaccines, presenting a new opportunity to target previously challenging diseases. Although the concept of mRNA drugs was envisioned over 30 years ago (Wolff et al., 1990), they were considered too unstable and immunotoxic for clinical use (Weng et al., 2020). Nonetheless, research into the chemical modifications of mRNA has shown that it can be used as an effective therapeutic agent. Moreover, mRNA offers distinct advantages over traditional drugs (Sahin et al., 2014). Compared to DNA technology, mRNA avoids the risk of genomic integration, circumvents the need to enter the nucleus, and has a transient activity profile, desirable in many gene therapy applications. mRNA vaccines can also be developed rapidly, can produce high quantities of antigen with relatively low dosages, and are safer and more readily produced at scale than traditional vaccines. Such benefits have been showcased in the first clinically approved mRNA vaccines against SARS-CoV-2 (Dolgin, 2021a; Kis et al., 2021).

The use of unmodified mRNA as a therapeutic agent is presented with several challenges and risks. Exogenously delivered mRNA is intrinsically immunogenic, triggering several innate immune sensing pathways, which leads to the production of inflammatory cytokines and suppression of cellular translation, undesirable for the production of the therapeutic protein (Tatematsu et al., 2018). Although the immunostimulatory nature of RNA could provide adjuvant activity for vaccinations, the translational inhibition and directed degradation caused by unmodified exogenous mRNAs mitigate their success (Morais et al., 2021). Other therapeutic strategies employing mRNAs, such as protein replacement therapy or regenerative therapy, are even less amenable to the strong stimulation of the immune system. The short half-life of mRNA, owing to its instability to degradation by ribonucleases, further obstructs the therapeutic application of mRNAs, limiting the protein production possible by delivered drugs. Improving both the lifespan and the translational efficiency of mRNA, in addition to removing its immune-activating nature, is thus necessary for successful therapeutics.

These technical challenges have been met by the development of mRNA modifications. Natural RNA contains many types of modifications, hundreds of which have been characterized (Boccaletto et al., 2018; Nachtergaele and He, 2018). Additionally, it has long been known that various viruses and bacteria decorate their genetic material with modifications to evade immune recognition by their host. With this motivation, several modified nucleotides have been incorporated during the *in vitro* transcription of RNA to make a synonymous modified transcript. Prominent among these substitutions is the replacement of uridine with pseudouridine (Ψ) and its methylated analog N1-methyl-pseudouridine (m^1^Ψ), which have been shown to dramatically reduce the stimulation caused by transcripts carrying these modified nucleotides (Dolgin, 2021b; Morais et al., 2021). Other work focusing on the translational capacity of mRNA have yielded longer-lasting, more highly translated transcripts through both nucleotide substitutions as well as targeted modifications of the 5′-cap and poly(A) tail, important protective structures against mRNA degradation. This enhancement has been attributed to a combination of increased resistance to exonucleases, decreased immune-triggered repression of translation, and greater rates of initiation, giving rise to much more effective protein production per transcript, enabling the burgeoning field of mRNA therapeutics. In this Review, we provide an overview of mRNA modifications relevant to mRNA therapeutics, as well as the current state of modified mRNA in clinical and preclinical studies.

## Overview of mRNA Therapeutics

Conceptually, mRNA therapeutics relies on the delivery of a synthetic transcript and subsequent translation of the encoded pharmacologically active protein product (Sahin et al., 2014). They are typically designed to be similar to natural mRNA, being able to harness the intracellular translational machinery in a functionally analogous or identical way. Natural mRNA is generally single-stranded, containing a coding sequence (CDS) which is translated to the protein product, flanked on either side by untranslated regions (UTRs). The 5′-end of mRNA in eukaryotes is marked with a 5′-cap, a modified 7-methyl-guanosine (m^7^G) residue, which modulates mRNA stability and lifespan (Charenton and Graille, 2018). Ribosomal translation typically is also cap-dependent, beginning with the association of eukaryotic initiation factor eIF4E to the transcript, after which the remainder of the translational machinery assembles and translates the encoded protein (Jackson et al., 2010). At the 3′-end, a chain of adenosine residues termed the poly(A) tail buffers against 3′ degradation and further regulates mRNA stability.

The synthesis of mRNA drugs is predominantly achieved by *in vitro* transcription (IVT) from a DNA template, using T3, T7, or SP6 polymerase in the presence of cap precursor and free nucleoside triphosphates. The transcript can alternatively be capped and polyadenylated post-IVT to produce a functional mRNA (Weissman, 2015; Muttach et al., 2017). During these stages, mRNA modifications can be introduced enzymatically through the incorporation of modified nucleotides and cap analogs in the reaction mixture (as discussed below). After purification of the newly synthesized mRNA, it is delivered to target cells to produce the pharmacologically active protein product, which is post-translationally modified and processed naturally. Substantial research has gone into delivering mRNAs, given their large molecular weight and highly negatively charged nature (Kowalski et al., 2019; Hou et al., 2021). In some applications, including cancer immunotherapy and stem cell therapy, mRNA can be electroporated into cells *ex vivo*, after which the transfected cells can be returned to the patient. More commonly, mRNA is encapsulated in a shell of neutrally or positively charged lipids, termed a lipid nanoparticle (LNP), which is endocytosed and promotes the release of the mRNA drug into the cytosol. A wide variety of LNPs has been designed to shield mRNAs from degradation, enhance cell transfection, and facilitate endosomal escape, resulting in overall increased delivery efficiency in preclinical models and demonstrating clinical success in the SARS-CoV-2 vaccines (Hou et al., 2021).

Once the mRNA drug reaches the site of interest, it begins producing of the desired protein, which can be used in a variety of therapeutic ways (Sahin et al., 2014). mRNA vaccines encode an antigenic-protein to stimulate the immune system. The vaccine can either be directly administered, through injection to intradermal, intramuscular, subcutaneous, and other locations; alternatively, *ex vivo* transfection of professional antigen-presenting cells, especially dendritic cells (DCs), has shown promise in treatments against cancer as a form of cell therapy. In either case, the translated protein is used to prime T cells and B cells in order to elicit protective immunity. Self-amplifying mRNAs, containing positive-sense RNA viral sequences that allow the mRNA to replicate, have also been tested for use in mRNA vaccines in order to increase the effective dose size and enable greater protein production (Bloom et al., 2021). Alternatively, mRNA can be used in protein replacement therapy to supplement the deficiency of a necessary protein or in regenerative medicine and gene therapy, reprogramming and gene-editing cells in order to restore function to target tissues and organs. The use of mRNA for remodeling otherwise untreatable tissues is promising for treating heart failure, neurodegeneration, etc. A more thorough description of mRNA therapeutic strategies is beyond the scope of this review and has been covered elsewhere (Sahin et al., 2014; Chandler, 2019; Zhang H.-X. et al., 2019; Damase et al., 2021).

## Immunogenicity of Exogenous mRNA

**FIGURE 1:**
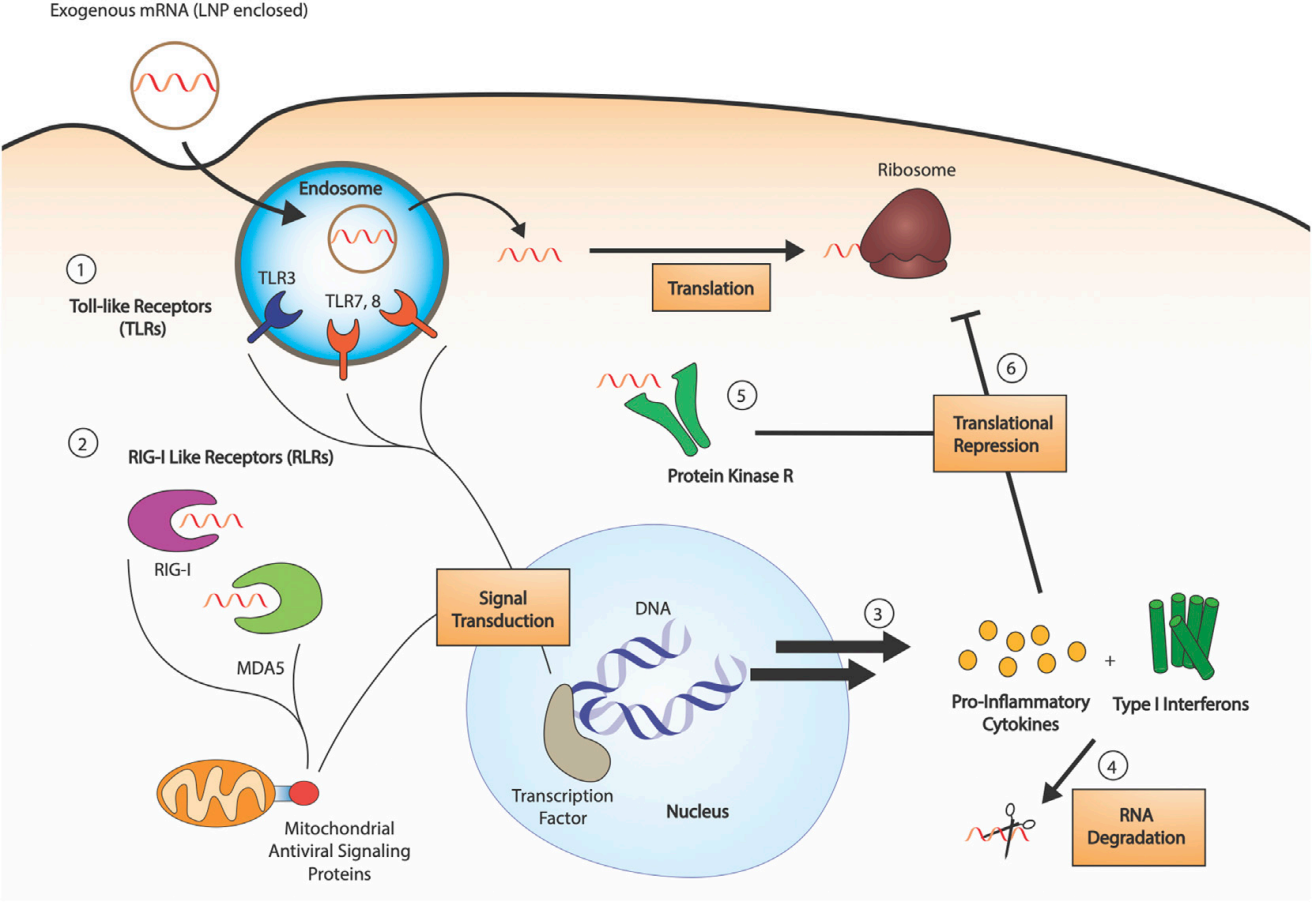
RNA sensing by the innate immune system. 1) RNA sensing Toll-like receptors (TLR3, TLR7, TLR8) are endosomal compartment receptors in sentinel cells, which activate upon late-endosomal acidification. Exogenous RNA is endocytosed by the cell, and pathogen associated molecular patterns are detected by the TLRs (dsRNAs, uridine-rich ribonucleosides, etc.). 2) RIG-I like receptors (RLRs) are cytosolic receptors present in all cell types. Both RIG-I and MDA5 are 5′-triphosphate dependent sensors, with some affinity for both dsRNA and ssRNA. Their activation leads to signal transduction through mitochondrial antiviral signaling proteins. 3) Innate immune detection of exogenous RNA leads to production of pro-inflammatory cytokines and type I interferons, which activate RNA degradation 4). 5) Protein kinase R (PKR) is a cytosolic sensor also involved in dsRNA sensing, the activation of which leads to phosphorylation of eukaryotic initiation factor eIF2α. 6) The combined action of produced cytokines and PKR leads to translational repression.

Exogenously delivered, unmodified IVT mRNA is an inherent immunostimulant, which poses a challenge to the efficacy of exogenously delivered mRNA drugs. Innate immune sensor detection of mRNA leads to inhibition of the cellular translational machinery and increased degradation of the mRNA, preventing effective protein production (Figure 1). Studies outlined below have revealed not only the underlying pathways relevant to mRNA-induced activation of the immune system but also that modifications can suppress the immune response. Pathogen-associated molecular patterns recognized by immune sensors have been studied; double-stranded RNA and double-stranded secondary structures have been highly investigated (Chen and Hur, 2022). Meanwhile, single-stranded mRNA recognition patterns are still not well understood. The following sections summarize key pathways in mRNA-associated immune regulation and how modifications help synthetic mRNA escape immune activation.

### Toll-Like Receptors

Toll-Like Receptors (TLRs) are a class of membrane-bound receptors present in sentinel cells of the immune system, such as dendritic cells and macrophages. Ten functional TLR family members have been identified in humans, four of which are responsible for the detection of nucleic acids: TLR9 recognizes unmethylated CpG DNA, TLR3 recognizes double-stranded RNA (dsRNA), and TLR7 and TLR8 recognize single-stranded RNA (ssRNA) (Kawasaki and Kawai, 2014). More specifically, TLR7 has shown to be activated by uridine-containing ribonucleosides, in addition to dsRNA (Diebold et al., 2006), whereas TLR8 responds to various ssRNA oligonucleotides and RNA degradation products. Nonetheless, the particular sequence preference of these ssRNA sensors is still unknown (Schlee and Hartmann, 2016). The four nucleic acid specific receptors are localized to the endosomal compartment and rely on endosomal acidification for activation (Figure 1). Upon TLR engagement, interferons (IFNs) and other inflammatory cytokines are secreted, causing the upregulation of a variety of interferon-stimulated genes (ISGs), including RNA degrading enzymes such as 2′-5′-oligoadenylate synthase (OAS) and RNase L (Anderson et al., 2011).

Various nucleotide modifications have been shown to be impactful in evading TLR activation. The replacement of all uridine residues with modified nucleotides, including pseudouridine (Ψ) and 2-thiouridine (s^2^U), was shown by Karikó and coworkers to ablate the TLR immunogenicity of IVT mRNA (Karikó et al., 2005). Transcripts containing these modifications had decreased inflammatory signaling, corresponding to an enhanced translational capacity. Later work demonstrated that N^1^-methyl-pseudouridine (m^1^Ψ) substitution exhibited an even better performance, attributed to decreased activation of TLR3 compared to other modifications (Andries et al., 2015). Other modifications, such as 5-methylcytidine (m^5^C), 5-methyluridine (m^5^U), and N^6^-methyladenosine (m^6^A), have also been shown to have some immunosuppressive effects on TLR activity (Lou et al., 2021). Altogether, nucleotide replacement effectively suppresses TLR-associated immune signaling.

### Retinoic Acid-Inducible Gene I Like Receptors

The retinoic acid-inducible gene I (RIG-I) like receptor family is a class of cytosolic pattern recognition receptors expressed in all cell types (Rehwinkel and Gack, 2020). This family consists of two primary receptors: the namesake RIG-I and melanoma differentiation-associated protein 5 (MDA5) (Figure 1). The two sensors are primarily associated with the detection of dsRNA: RIG-I senses short dsRNA segments containing 5′-triphosphates (Hornung et al., 2006), whereas MDA5 preferentially binds long dsRNAs. RIG-I can also detect 5′-triphosphate-containing ssRNAs, and the precise requirements for its activation are still being determined. MDA5 also has been suggested to detect the RNA of some ssRNA viruses, potentially due to the formation of secondary structures (Schlee, 2013). Despite the expanding understanding of their ligand range, the RIG-I-like receptors are a major part of the interferon response to RNA.

As a 5′-triphosphate is important for activation of RIG-I, the addition of a synthetic cap to IVT mRNA plays a critical role in evading RIG-I detection. The installation of an N^7^-methylguanosine (m^7^G) residue to the 5′ end of triphosphate mRNA decreases the RIG-I-dependent IFN secretion by synthetic transcripts (Hornung et al., 2006). Furthermore, modified nucleotide substitutions can also play an inhibitory role in RIG-I signaling, with Ψ, s^2^U, and 2′-O-methyluridine all reducing the total inflammatory cytokine-induced by 5′-triphosphate-containing mRNA (Karikó et al., 2008).

However, capping and nucleotide replacement are unable to fully abrogate the RIG-I dependent response to 5′-triphosphate mRNA (Schuberth-Wagner et al., 2015). Structural studies on RIG-I binding revealed that the receptor can accommodate the presence of an m^7^G moiety without drastic disruption of its triphosphate recognition (Devarkar et al., 2016). On the other hand, methylation of the 5′-most nucleotide of capped mRNA strongly interferes with RIG-I binding, and methylation of the second nucleotide is also implicated in decreasing RIG-I’s activation. Indeed, higher eukaryotic mRNA generally contain a 2′-O-methylated first nucleotide, termed a cap-1 structure in contrast with the unmethylated cap-0’s, and coronaviruses and poxviruses have been shown to employ cap one modifications to evade the innate immune system (Daffis et al., 2010). MDA5, although also 5′-triphosphate dependent, induces IFN production even in the presence of cap-0 structures but is inactive in the presence of cap-1 mRNA (Züst et al., 2011). Moreover, IFIT1, a major interferon induced gene, further recognizes 5′-triphosphates and cap-0 mRNA, inhibiting translation by competing with eIF4E, a cap-binding translation initiation factor (Habjan et al., 2013). Cap-1 demonstrated decreased IFIT1 binding activity, further assisting immune system evasion of modified mRNAs.

### Protein Kinase R and eIF2α Phosphorylation

Protein kinase R (PKR) is an interferon-induced protein kinase (Figure 1), capable of being activated by either dsRNA (>33 bp) or ssRNA containing an exposed 5′-triphosphate (Nallagatla and Bevilacqua, 2008). Upon activation and autophosphorylation, PKR then phosphorylates the α-subunit of eIF2, the GTP-dependent translation initiation factor responsible for mediating binding of the first aminoacyl-tRNA (Met-tRNA) to the ribosome. Phosphorylation enhances the binding affinity of eIF2 for its GTP exchange factor, eIF2B, causing sequestration which results in impaired translation. Substitution of uridine using Ψ, s^2^U, 2′-dU, and other modifications are able to inhibit PKR signaling (Anderson et al., 2010). Notably, m^1^Ψ exhibited strong repression of PKR activation, outperforming Ψ and other modifications (Svitkin et al., 2017).

### Future Directions

Much progress has been made in understanding the immune mechanisms and modifications relevant to mRNA therapeutics. Nonetheless, multiple confounding factors have complicated the research. Indeed, dsRNA contaminants cause residual stimulation of multiple innate immune sensors, and multiple purification methods have been developed to counteract this, including HPLC (Karikó et al., 2011) and RNase III digestion (Foster et al., 2019). Differences in the manufacturing process, such as the ratio of modified to unmodified nucleotides present in the IVT reaction mixture, also lead to differences in dsRNA byproduct formation (Nelson et al., 2020). Additionally, the immune response to mRNAs is highly dependent on the system under study, with variable results depending on target cell type, temperature, etc. (Uchida et al., 2015; Li et al., 2016) For example, whereas RNAs containing both m^1^Ψ substitution for uridine and m^5^C for cytidine had a higher translational yield *in vitro*, mRNA with only m^1^Ψ demonstrated higher performance *in vivo* in mice (Andries et al., 2015). As such, more investigation with standardized conditions and preparation is necessary to comprehensively understand the effects of mRNA modifications on immune responses.

## Stability and Translational Efficiency of mRNA

**FIGURE 2:**
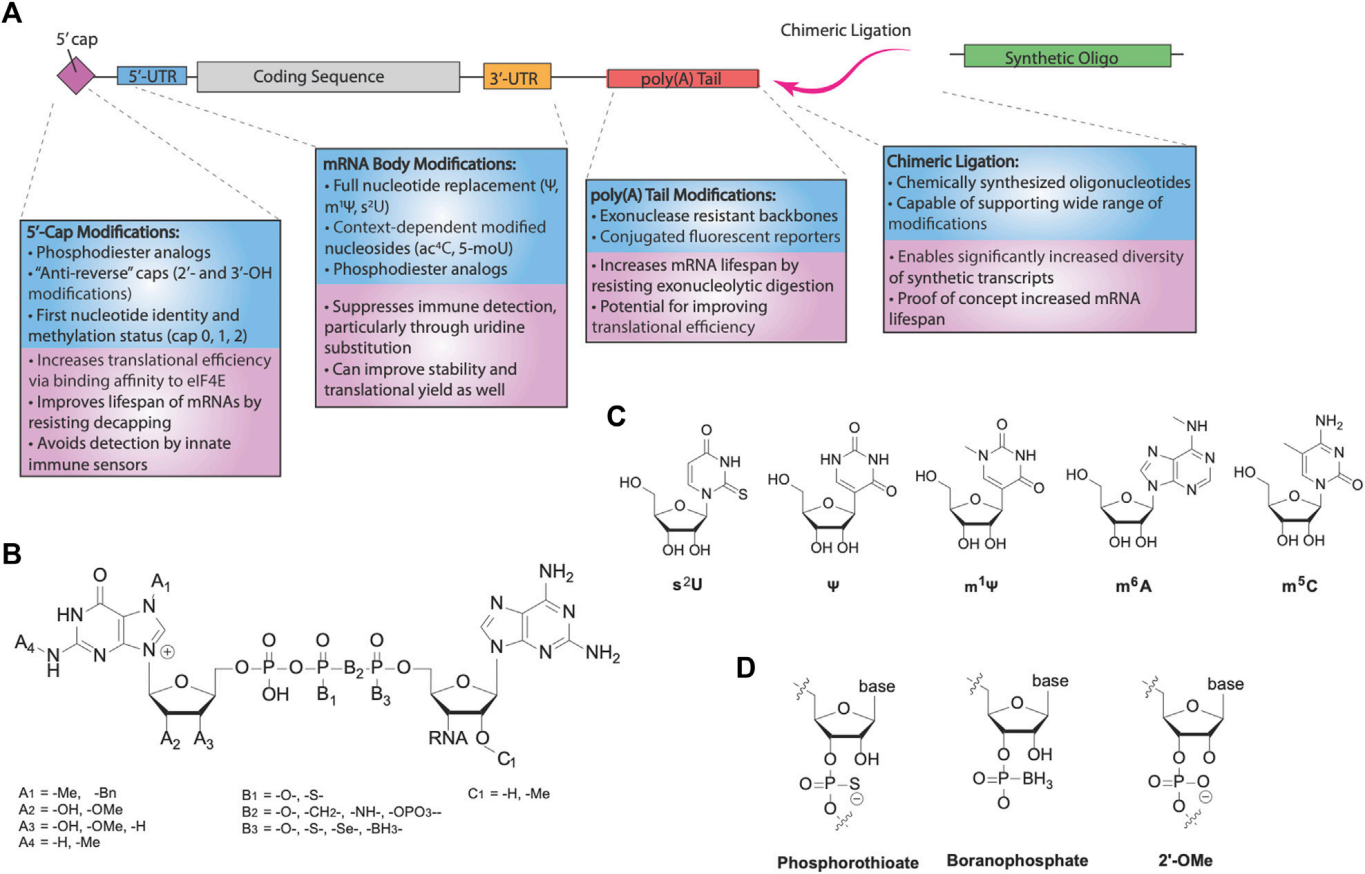
RNA modifications for mRNA therapeutics. **(A)** Categories of different modifications for mRNA. Modification of the cap and nucleotide substitution of the mRNA body are important for innate immune avoidance. Translational efficiency and mRNA stability are further modulated by various modifications, *via* increased eIF4E binding and reduced hydrolysis by nucleases. Additionally, chimeric ligation is a separate class of modification enabling incorporation of highly modified synthetic oligonucleotides, forming chimeric mocRNAs. **(B)** Chemical structure of 5′-caps. Eukaryotic caps are typically modified on the first base (A’s), triphosphate (B’s), or second base (C’s). **(C)** Common modified bases used for modification of mRNA. 2-thiouridine (s^2^U), pseudouridine (Ψ), and N^1^-methylpseudouridine (m^1^Ψ) are uridine substitutes, whereas N6-methyladenosine (m^6^A) is an adenosine substituent and 5-methylcytosine (m^5^C) is a cytosine substituent. **(D)** Common backbone modifications used for modification of mRNA. The phosphate backbone and 2′-OH are frequently modified.

The effectiveness of mRNA therapeutics depends highly on the amount of protein that can be produced from a given transcript. This translational yield is dependent both on the lifespan of mRNA as well as the rate of translational initiation. Years ago, significant doubt arose over the capability of mRNA as a drug, primarily due to its instability from both immune-induced degradation and its intrinsically shorter half-life from other therapeutic modalities. However, chemical modifications of mRNA targeted at decreasing its susceptibility to enzymatic degradation have been able to greatly increase the lifetime of IVT RNAs (Figure 2A). Additionally, the same modifications also affect the translational efficiency of delivered transcripts, leaving further potential for increasing the protein yield of mRNA drugs. Here, we review various modification strategies in order to improve the translational capacity of IVT mRNA.

### The 5′-Cap

The degradation of mRNA is mediated primarily through two pathways: 5′ → 3′ and 3′ → 5′ degradation. In the 5′ → 3′ pathway, decapping of the 5′-end *via* cleavage of the α-β phosphodiester bond by the Dcp1/2 decapping complex precedes exonucleolytic degradation of the mRNA, primarily by the ribonuclease Xrn1 (Charenton and Graille, 2018). Thus, the stability of the 5′-cap is essential for controlling the lifespan of mRNA. The 5′-cap also exerts an effect on translational yield through modulating translational efficiency (Jackson et al., 2010). Translation is typically rate limited by the initiation step, a generally cap-dependent process reliant on binding of initiation factor eIF4E to the 5′-cap. Given its significance in both translational efficiency and mRNA stability, optimizing the 5′-cap of mRNA is crucial for designing more effective mRNA drugs (Figure 2A).

Two strategies are generally employed to cap synthetic mRNAs (Muttach et al., 2017). Recombinant viral capping enzymes, such as the vaccinia virus capping enzyme (VCE), can be used in conjunction with a methyltransferase in the presence of GTP and the 5′-triphosphate IVT mRNA to add a cap-1 structure. More commonly, however, co-transcriptional capping can be performed using a cap dinucleotide in the presence of the IVT polymerase mixture. The 3′-OH of the cap dinucleotide nucleophilically attacks the α phosphate of the next nucleotide, and elongation by the polymerase continues onwards. However, due to the similarity between the two 3′-OH’s present in the dinucleotide, capping with unmodified dinucleotides results in the wrong orientation at least half of the time, reducing the translational efficiency of the product mRNAs (Stepinski et al., 2001). To address this, Rhoads and others designed anti-reverse cap analogs (ARCAs), modified dinucleotides containing a 3′-O, 7′-dimethylguanosine or 3′-deoxy-7-methylguanosine, preventing incorrect incorporation into synthetic transcripts and more than doubling their translational efficiencies relative to unmodified cap dinucleotides. Alternative modifications were also shown to enforce the correct orientation, including 2′-O-methylation (Jemielity et al., 2003) and N^7^-benzyl-N^2^-methyl- dual modification (Grudzien et al., 2004). In all, the use of ARCAs allows for improved synthesis and function of mRNA drugs.

A series of ARCAs have since been synthesized and explored to improve the performance of synthetic transcripts while maintaining the anti-reverse function of these analogs (Figure 2B). For example, tetraphosphate analogs of the first-generation ARCA dinucleotides improved the translational yield of mRNAs, associated with the higher binding affinity for eIF4E (Muttach et al., 2017). Surprisingly, pentaphosphate counterparts did not recapitulate this trend, with a lower translational efficiency despite even higher binding affinities for eIF4E. This effect was attributed to slower release kinetics of eIF4E after initiation, indicating the strength of eIF4E binding does not directly imply higher translational efficiency. Meanwhile, modifications targeted towards improving IVT mRNA stability to decapping focused on altering the phosphodiester moiety. Grudzien et al. (2006) demonstrated that Dcp1/2 acts primarily on the α, β phosphodiester bond and replacement of the bridging oxygen with a methylene group (-CH_2_) blocked 5′ → 3′ degradation, albeit with some cost towards translational efficiency. Motivated by evidence that phosphorothioate modification of the mRNA backbone could also increase stability, later generations of cleavage-resistant caps used modifications of either the α or β phosphates with a phosphorothioate (Grudzien et al., 2007). Phosphorothioate modified caps yielded higher translational efficiencies than unmodified ARCAs, while simultaneously greatly improving the half-life of synthetic transcripts. Polysome profiling studies revealed that a greater rate of initiation is responsible for the increased translation rate, and phosphorothioate cap analogs have also been demonstrated to be effective in dendritic cells and *in vivo* in mice for vaccination and immune system priming (Kuhn et al., 2010). 1,2-dithiodiphosphates were also tested and demonstrated even higher stability profiles than phosphorothioate caps (Strenkowska et al., 2016).

A slew of other analogs have been explored as well, including phosphorothiolate (Wojtczak et al., 2018), phosphoroselenoate (Kowalska et al., 2009), boranophosphate (Kowalska et al., 2014), imidodiphosphate modified caps (Rydzik et al., 2012), etc. (Warminski et al., 2013; Shanmugasundaram et al., 2016; Dülmen et al., 2021; Wojcik et al., 2021) Locked nucleic acid (LNA) caps have also been investigated, in which the ribose is locked in an C3′-endo conformation by a bridging methylene group between the 2′ oxygen and 4′ carbon (Kore et al., 2009). Although LNAs have primarily been used in oligonucleotides, mRNAs capped by an LNA analog have recently been demonstrated to have increased translational efficiency and stability (Senthilvelan et al., 2021). Given the promise many of these modifications have demonstrated in *in vitro* experiments, the optimization of capped mRNAs using these analogs *in vivo* and in clinical applications holds promise for even more effective future drugs.

### mRNA Body Modifications

Nucleoside and backbone modifications of the DNA encoded mRNA body are critical to enhance the protein production of mRNAs (Figures 2A,C,D). Ψ and m^1^Ψ are the most widely used body modifications for mRNA therapeutics. When incorporated as 100% replacement for U, they significantly increase the translational efficiency of mRNAs by turning off the innate immune-triggered eIF2α phosphorylation-dependent inhibition of translation (Karikó et al., 2008; Svitkin et al., 2017). Moreover, in comparison with Ψ, m^1^Ψ showed further enhancement of translational capacity, which has been linked to its capability of increasing ribosome density on the mRNA. Specifically, the additional methyl group on m^1^Ψ blocks hydrogen bonding at the N1 position, despite resulting in ribosome pausing, dramatically increasing the ribosome loading per mRNA (Svitkin et al., 2017), which may potentially increase translation initiation and prevent mRNA from entering degradation pathways. Thus, full-length body modifications using immunosuppressive and translation-enhancing modified nucleosides can generate mRNA drugs with greatly improved translational capacity.

Earlier attempts of backbone modification *via* IVT incorporation of phosphorothioates showed successful translation in reconstituted *E. coli* *in vitro* translation system (Ueda et al., 1991; Tohda et al., 1994). A recent study further uncovered that introduction of phosphorothioates to the 5′-UTR at either cytidine or both cytidine and uridine increases translational efficiency *via* faster initiation, even at the expense of elongation processivity (Kawaguchi et al., 2020). Other familiar modifications, including m^6^A and s^2^U, can increase RNA stability by decreased activation of the 2′-5′-oligoadenylate synthetase system (OAS), an interferon associated pathway that leads to RNase L activation (Anderson et al., 2011). In addition, some modifications have been revealed to exert a context-dependent effect on mRNA translational yield. The first nucleotide after the 5′-cap appears to play an important role in protein production (Sikorski et al., 2020). Adenosine and m^6^A residues at this site demonstrate higher translational yields, and 2′-O-methylation of the first nucleotide modulates protein production based on the identity of the first nucleotide. N^4^-acetylcytidine (ac4C) also increases transcript stability and translational yield in a position specific manner, increasing the speed of mRNA decoding when positioned at a wobble site (Arango et al., 2018). Another study revealed that 5-methoxyuridine (5-moU) is also capable of increasing mRNA stability, though further research is required to unravel the underlying mechanism of its enhancement (Li et al., 2016). In all, the diversity of potential chemical modifications gives substantial promise for even better-performing mRNA therapeutics.

### The poly(A) Tail

The poly(A) tail is a chain of adenosine residues at the 3′-end of mRNA, which buffers it from degradation in a length-dependent fashion. Poly(A) shortening is catalyzed by the Pan2-Pan3 deadenylation complex, preceding both 3′ → 5′ and 5′ → 3′ degradation. Moreover, the tail and cap of actively translated mRNA interact, providing a mechanism by which the poly(A) tail can affect translational efficiency (Gallie, 1991; Goss and Kleiman, 2013). Although earlier works attempting to modify the poly(A) tail were met with disappointment (Rabinovich et al., 2006), more recent studies have indicated that there is still potential for improving stability and translational yield through poly(A) tail modifications (Figure 2A). Indeed, initial efforts to modify the poly(A) tail employed 3′-deoxyadenosine (cordycepin) or 8-aza-adenosine, which were shown to stabilize mRNA similarly to lengthening of the poly(A) tail, but were unable to outperform them in translational assays. Nonetheless, phosphorothioate modification of the poly(A) tail was able to exhibit increased stability and translational yield in some systems (Strzelecka et al., 2020). Boranophosphate substitution has also been tested, but underperformed compared to phosphorothioate functionalization. Interestingly, attachment of sulforhodamine B (SRB), a fluorescent small-molecule label, using click chemistry with incorporated 2′-azido-2′-dATP was able to substantially increase translational efficiency, though the mechanism of such enhancement has yet to be determined (Anhäuser et al., 2019). In all, despite the rather limited exploration of poly(A) tail modifications, future research into the poly(A) tail can likely further improve mRNA therapeutics.

### Chimeric RNA

Recently, our group has demonstrated the generation of chimeric mRNAs, formed by the enzymatic ligation of an IVT synthesized mRNA transcript with a chemically synthesized oligonucleotide (Aditham et al., 2022) (Figure 2A). Termed mRNA-oligonucleotide conjugated RNA (mocRNA), this platform presents a novel method of circumventing translational restrictions on incorporating modified nucleotides and expands the possible space of synthetic transcripts for therapeutics. In our work, nuclease-resistant oligonucleotides were ligated to the poly(A) tail, resulting in 3–10 folds higher expression in human HeLa cells and rat primary neurons. The programmable and modular nature of mocRNAs enabled engineering mRNAs without interfering with the coding region. Future work into diversifying the ligated oligonucleotides will likely further illustrate the potential of chimeric RNAs.

## Clinical and Preclinical Examples of Modified RNA

Various candidate mRNA therapeutic drugs have been examined both preclinically and clinically in the past years and have been reviewed extensively. Here, we highlight a number of these which employed modified mRNAs.

### Vaccines

A number of vaccines based on modified mRNA have been developed (Zhang C. et al., 2019). Most prominent of the modified mRNA vaccines are those against SARS-CoV-2, advanced by Moderna (mRNA-1273) and BioNTech in partnership with Pfizer (BNT-162b2). Both vaccines encode the prefusion conformation of spike glycoprotein using N^1^-methyl-pseudouridine encoding mRNAs containing a 5' cap-1 (Corbett et al., 2020; World Health Organization 2020). mRNA modifications proved to be critical for the success of these vaccines, with similar products with unmodified mRNAs underperforming expectations (Morais et al., 2021; Nance and Meier, 2021). Moreover, the use of mRNA as a platform for a vaccine during the COVID-19 pandemic proved advantageous, owing to the rapid development and manufacturing speed of mRNA (Kis et al., 2021). Indeed, both vaccines were able to be produced within 10 months after the sequencing of the SARS-CoV-2 genome and proved to be over 90% effective (Polack et al., 2020; El Sahly et al., 2021). mRNA is also easily adaptable to new strains and mutations. The prefusion spike protein encoded in the aforementioned vaccines uses missense mutations at two loci in the original sequence to enforce the proper immunogenic conformation. As the SARS-CoV-2 virus continues to evolve, the adjustability of the mRNA vaccine platform will be critical.

Various influenza virus vaccines using modified mRNA have also been under development (Dolgin, 2021c). A vaccine candidate against H10N8 and H7N9 entered phase I trials in 2015 (Feldman et al., 2019), and two other candidates (mRNA-1010 and PF-07252220) entered phase I trials in late 2021. mRNA-1010 is a quadrivalent vaccine against the H1N1, H3N2, Yamagata, and Victoria strains, whereas the PF-07252220 is currently a monovalent vaccine, which is planned to be combined into a bivalent or quadrivalent product in the future. A slew of other mRNA influenza vaccine candidates have also undergone preclinical testing. The advent of mRNA vaccines against the flu is particularly exciting, as traditional flu vaccines are often ineffective and inconsistently manufactured (Wu et al., 2017). Additionally, due to constraints on the time necessary to develop traditional vaccines, the yearly influenza vaccines are often disappointingly ineffective. On the other hand, mRNA can easily be adjusted to encode antigens for the precise strain of influenza relevant, and its scalability bypasses the error-prone egg-based method for producing traditional vaccines. Altogether, the growing interest in modified mRNA vaccines holds promise for flu vaccinations in the future.

Clinical trials have also been initiated for a number of other diseases, which have posed a challenge for traditional vaccines. Phase III trials for a modified mRNA vaccine against cytomegalovirus (CMV) began late in 2021, after promising early results (John et al., 2018). Phase I trials of a modified mRNA vaccine against HIV have also recently begun in January 2022. Preclinical studies have also been performed for modified mRNA vaccine candidates against Ebola (Meyer et al., 2018), Zika (Pardi et al., 2017) human metapneumovirus (hMPV) (Shaw et al., 2019), etc.

Due to the highly polymorphic nature of cancer profiles, effective therapeutic vaccines against cancer often require individualization. Modified mRNA has been used in multiple preclinical and clinical applications against cancer, primarily in direct vaccine injections. LNP encapsulated modified mRNAs encoding bispecific antibodies (Stadler et al., 2017), cytokines (Hewitt et al., 2019; Kranz et al., 2019; Vormehr, 2019), or chimeric antigens (Foster et al., 2019) have been investigated. A full coverage of mRNA in cancer therapeutics can be found in other reviews (Beck et al., 2021; Miao et al., 2021).

### Replacement and Gene Therapy

Protein production from mRNA has also been investigated as a tool for replacement and gene therapies. As opposed to DNA-based therapies, modified mRNAs demonstrate a pulse-like expression profile and do not risk genomic integration - problems that hindered previous efforts in such therapeutic approaches.

Potential use of modified mRNA as a vector for reprogramming and regenerative medicine was first demonstrated in 2010, when Warren and others used repeated transfections of reprogramming factor-encoding mRNAs to generate pluripotent stem cells (iPSCs) from fibroblasts with relatively high efficiency (Warren et al., 2010). These mRNAs were modified with m^5^C and Ψ substitutions for C and U, respectively, reducing the innate immune response against ectopic mRNA, and improving viability of targeted cells. Interestingly, evidence suggests that some residual inflammatory signaling may actually play a role in assisting reprogramming (Lee et al., 2012), but the presence of immunosuppressive modification nonetheless helped avoid translational silencing of the transcripts and overstimulation of the immune system. Indeed, the repeated transfection regime was only made possible by suppression of the innate immune system, indicating an essential role for mRNA modifications. The use of mRNA reprogramming for iPSC generation therapeutically has been covered elsewhere (Shi et al., 2017; Warren and Lin, 2019).

In addition to reprogramming, modified mRNAs have significant therapeutic potential in regenerative medicine, especially in organs and tissues with little regenerative capacity. In the heart, VEGF-A expression from modified mRNA resulted in healthy regeneration of cardiac vasculature after myocardial infarction in mice and swine (Zangi et al., 2013; Carlsson et al., 2018). In contrast, DNA-based expression was prolonged and resulted in edema and death. Additionally, Phase II a trials of a modified mRNA encoding VEGF-A have also been performed in patients with coronary artery disease, with generally positive results (Anttila et al., 2020). Expression of other proteins, including PKM2, FSTL1, and IGF-1 were used to promote cell survival and cardiomyocyte regeneration *in vivo*, improving general pathophysiology (Kaur and Zangi, 2020). VEGF-A mRNA has also been tested for the treatment of type II diabetes, yielding enhancements in skin blood flow in a phase I trial (Gan et al., 2019). Thus, mRNA holds potential as a platform for VEGF-A induced revascularization.

Modified mRNAs have been used in a variety of other regenerative medicinal applications. Attempts to prevent cell death in neuronal tissue after ischemic attack (Fukushima et al., 2021), to induce regeneration following liver damage (Rizvi et al., 2021), etc. have been successful preclinically. Delivery of gene editing enzymes through the expression of modified mRNAs have also presented an opportunity for gene therapies, circumventing many previous challenges of such strategies (Zhang H.-X. et al., 2019). Finally, modified mRNAs may also be used for direct replacement therapies for deficient proteins, including surfactant protein B (SP-B) (Kormann et al., 2011), arginase 1 (ARG1) (Asrani et al., 2018), cytochrome c oxidase (SCO2) (Miliotou et al., 2021), etc. In all, modified mRNA-based gene therapies provide an opportunity to treat many previously challenging diseases.

## Conclusion and Perspectives

With the increasing popularity and maturation of mRNA therapeutics, significant progress has been made in understanding the role of mRNA modifications in attuning their immunogenicity, stability, and translational efficiency. Nucleotide substitutions and cap modifications play important parts in reducing innate immune sensing of IVT mRNA. Furthermore, modifications promoting translational initiation increase the translational yield of modified RNAs, and modifications resisting degradation by decapping or deadenylation increase the half-life of mRNA drugs for more sustained expression. Research into mRNA modifications has yielded multiple candidate mRNA therapeutics undergoing clinical or preclinical trials, as well as effective SARS-CoV vaccines.

However, mRNA modifications have yet to be fully employed in therapeutics. The diversity of known modifications has not been reflected in current mRNA drug candidates, which primarily focus on substitutions of uridine with N^1^-methyl-pseudouridine and cap methylation state. Given the evidence that phosphodiester modifications, labeling of the poly(A) tail, and other nucleoside substitutions are capable of increasing the stability and translational yield of mRNA, many optimizations can likely be made to future mRNA therapeutics. Indeed, in addition to altering the necessary dosage of mRNA drugs, modifications could also foreseeably increase their shelf-life, which is currently one of the major criticisms of their practicality. Nonetheless, the sensitivity and context-dependence of modified mRNAs’ performance requires further efforts to parse the precise effects of mRNA modifications on immunosuppression, translation, and stability.

Additionally, further insight into biological pathways relevant to mRNA therapeutics may motivate the targeted use of modifications. The importance of poly(A) tail modifications on translational initiation have yet to be fully understood and leaves room for potential improvements. Similarly, advances and new techniques in sequencing technology have enabled the discovery of new therapeutically relevant modifications. Surveying the effects of these new modifications and the mechanisms underlying them could lead the way to even more effective therapeutics. Finally, on a more cautionary note, further research into the long-term effects of highly modified mRNAs (including downstream byproducts of modified bases) are desired for the safe use in mRNA therapeutics. Nonetheless, given recent advances in modified mRNAs, future mRNA therapies will likely be shaped by progress in RNA modifications and have unlimited potentials in treating other diseases beyond mRNA vaccines.
